# High expression of ZFP36L2 correlates with the prognosis and immune infiltration in lower-grade glioma

**DOI:** 10.3389/fgene.2022.914219

**Published:** 2022-07-15

**Authors:** Min Zhou, Jinquan Li, Cheng Chen

**Affiliations:** ^1^ College of Medicine, Wuhan University of Science and Technology, Wuhan, China; ^2^ Wuhan Asia Heart Hospital, Wuhan, China

**Keywords:** ZFP36 ring finger protein like 2 (ZFP36L2), lower-grade gliomas, immune infiltration, prognostic value, TCGA

## Abstract

**Background:** The ZFP36 Ring Finger Protein Like 2 (ZFP36L2) is an RNA-binding protein that regulates gene expression at post-transcriptional level. However, the clinical significance and prognostic value of ZFP36L2 in lower-grade glioma (LGG) remain unclear.

**Method:** ZFP36L2 expression was investigated using public datasets and the prognostic merit of ZFP36L2 with LGG patients was further evaluated. The correlation between the genetic alteration of ZFP36L2 and its mRNA expression was accessed via cBioPortal. Additionally, the prognostic value of the ZFP36L2 methylation levels in LGG was evaluated by MethSurv. The potential biological role of ZFP36L2 in LGG was identified by performing functional analyses. We also examined the correlation between ZFP36L2 expression and the immune infiltration. Finally, the predictive value of ZFP36L2 to immunotherapy was assessed.

**Result:** ZFP36L2 was highly expressed in LGG patients and overexpressed ZFP36L2 predicted poor clinical outcomes. We further identified ZFP36L2 as an independent prognostic factor. The methylation level of ZFP36L2 negatively correlated with the ZFP36L2 expression, and patients with low ZFP36L2 methylation had worse overall survival. The results of functional analysis indicated that ZFP36L2 was involved in multiple immune response-related pathways in LGG. Furthermore, high expression of ZFP36L2 was significantly and positively correlated with immune infiltration. Finally, we found that ZFP36L2 expression was positively correlated with the immune checkpoint PD-L1, and ZFP36L2 low expression cohort gained better benefit from immunotherapy.

**Conclusion:** Our findings demonstrate that ZFP36L2 is a potential biomarker for LGG, highlighting its potential as a therapeutic target in immunotherapy.

## 1 Introduction

Glioma is one of the most common primary malignant brain tumors, accounting for nearly 30% of all primary intracranial tumors, and 80% of all malignant intracranial tumors (Weller et al., 2015). Lower-grade gliomas (LGG) are classified as World Health Organization (WHO) grades II and III types according to the WHO system (Wesseling and Capper, 2018). Although there are a variety of treatments either alone or in combination for LGG, effective and reliable biomarkers that can guide specific treatment strategies are rare. Therefore, additional biomarkers are required to provide a basis for the diagnosis and treatment of LGG ((Wentworth et al., 2009; Sottoriva et al., 2013; Cuddapah et al., 2014; Kwon et al., 2015)).

ZFP36L2 is part of the RNA binding protein family that regulates cytoplasmic mRNA fate by directly binding to 3′-UTR AREs (Carballo et al., 1998; Brooks and Blackshear, 2013; Molle et al., 2013). Aberrant expressed ZFP36L2 has been found in most cancers. Early studies have reported that ZFP36 possessed anti-tumor function in ovarian, breast cancer and colorectal cancer (Jackson et al., 2006; Carrick and Blackshear, 2007; Chou et al., 2013; Suk et al., 2017). Conversely, Xing et al. showed that increased expressed ZFP36L2 due to altered super-enhancer promoted the cell aggressiveness of gastric cancer (Xing et al., 2019). Furthermore, Yonemori et al. revealed the positive correlation between ZFP36L2 and pancreatic ductal adenocarcinoma cells (Yonemori et al., 2017). But until now, the precise function and prognostic value of ZFP36L2 in lower-grade gliomas have not been elucidated.

In current study, we used integrated bioinformatic approaches to explore the potential mechanisms of ZFP36L2 involvement in glioma development and its potential as a prognostic biomarker for LGG. Our results suggest that ZFP36L2 expression is significantly higher in LGG and ZFP36L2 overexpression is a reliable independent prognostic factor for LGG. We also determined the correlation between ZFP36L2 expression and genetic changes. Further functional analysis reveals that ZFP36L2 is involved in multiple immune response-related pathways. Collectively, our study suggests that ZFP36L2 plays an important role in the tumor immune microenvironment and is predictive of immunotherapeutic response.

## 2 Materials and methods

### 2.1 Datasets collection

Publicly attainable gene expression and corresponding clinical annotations of LGG samples were collected from TCGA database, CGGA database and NCBI GEO database, and a total of 1208 patients were included for the further analysis, including GSE16011 (N = 284), TCGA-LGG (N = 530), CGGA-693 (N = 250) and CGGA-325 (N = 144). For pan-cancer dataset, TCGA TARGET GTEx (PANCAN, N = 19,131, G = 60,499) was downloaded from the UCSC database (Goldman et al., 2020), and further we extracted ZFP36L2 expression data in each sample. The level 3 HTSeq-FPKM data were transformed to TPM (transcription per million reads) for the following analyses. Patients with LGG were classified into low- and high-expression groups according to their median expression value of ZFP36L2.

### 2.2 Examination of ZFP36L2 independent prognostic value

To figure out whether ZFP36L2 is an independent biomarker in LGG, we used univariate and multivariate analyses. Several clinical factors were enrolled, including gender, age, grade, location and pathology. *p*-value less than 0.05 was considered as statistically significant.

### 2.3 Gene Set Enrichment Analysis

To reveal the underlying biological molecular changes related to ZFP36L2 expression, we divided LGG samples into ZFP36L2-high and -low group based on the expression of ZFP36L2. GSEA (Subramanian et al., 2005) was performed by comparing ZFP36L2-high cohort with -low cohort and ‘h.all.v7.4.symbols.gmt’ was selected as the background gene set. *p*-value <0.05 and False Discovery Rate (FDR) < 0.25 were considered as statistically significant.

### 2.4 Gene Set Variation Analysis

Gene Set Variation Analysis (GSVA) was utilized to reveal the different biological processes between high and low ZFP36L2 expression groups. The gene set ‘h.all.v7.4.symbols.gmt’, which was acquired from the MSigDB database (Liberzon et al., 2015), was selected as the background gene set.Gene Set Enrichment Analysis (GSEA).

### 2.5 ZFP36L2 genetic alteration and its prognosis analysis

cBioPortal Database was used to analyze the association between ZFP36L2 genetic alteration and its mRNA expression (Cerami et al., 2012). MethSurv online tool was used to explore the prognostic value of the ZFP36L2 methylation level in the TCGA-LGG cohort (Modhukur et al., 2018).

### 2.6 Immune infiltration analysis

The ESTIMATE algorithm (Yoshihara et al., 2013) was used to study the Immune Infiltration degree with distinct ZFP36L2 expression pattern. Tumor Immune Estimation Resource (TIMER) (Li et al., 2017) was used to evaluate the correlation between gene expression and the infiltration of various types of immune cells. We analyzed the relationship between ZFP36L2 expression and a variety of tumor-infiltrating immune cells online, including B cells, CD8^+^ T cells, CD4^+^ T cells, macrophages, neutrophils and dendritic cells. Furthermore, the CIBERSORT algorithm (Chen et al., 2018) was utilized to evaluate the attribution of ZFP36L2 expression on the immune cell infiltration of the tumor microenvironment.

### 2.7 Therapeutic response analyses

ImmuCellAI, a method that has the ability to predict the response of ICI therapy through analyzing the distribution and abundance of immune cells, especially T-cell subsets, was applied to clarify the potential linkages between ZFP36L2 and the therapeutic response of ICIs(Miao et al., 2020).

### 2.8 Western blot analysis

Total proteins were isolated using a protein extraction kit (Beyotime, Nantong, China) according to standard protocols. The antibodies used were: ZFP36L2 (1:500, 19005-1-AP, Proteintech), and GAPDH (1:1000, 60004-1-Ig, Proteintech). Horseradish peroxidase (HRP) -conjugated secondary antibodies (Proteintech) were used and protein bands were visualized and detected using an enhanced chemiluminescence system. GAPDH was used for normalization. The experiments were performed in triplicate.

### 2.9 Statistical analyses

Unpaired *t*-test was used to compare the expression levels of ZFP36L2 between different groups, and *p* < 0.05 was considered significant. The median expression level of ZFP36L2 was used to distinguish between the OS of patients with LGG. Survival curves are plotted using the Kaplan-Meier method, and the OS differences between the groups were evaluated using log-rank test; here as well, *p* < 0.05 was considered significant. All statistical analyses were performed using R (version 4.0.2), SPSS (version 26.0) and GraphPad Prism 8.0.

## 3 Results

### 3.1 Expression levels of ZFP36L2 across different types of tumor and normal tissues

**FIGURE 1:**
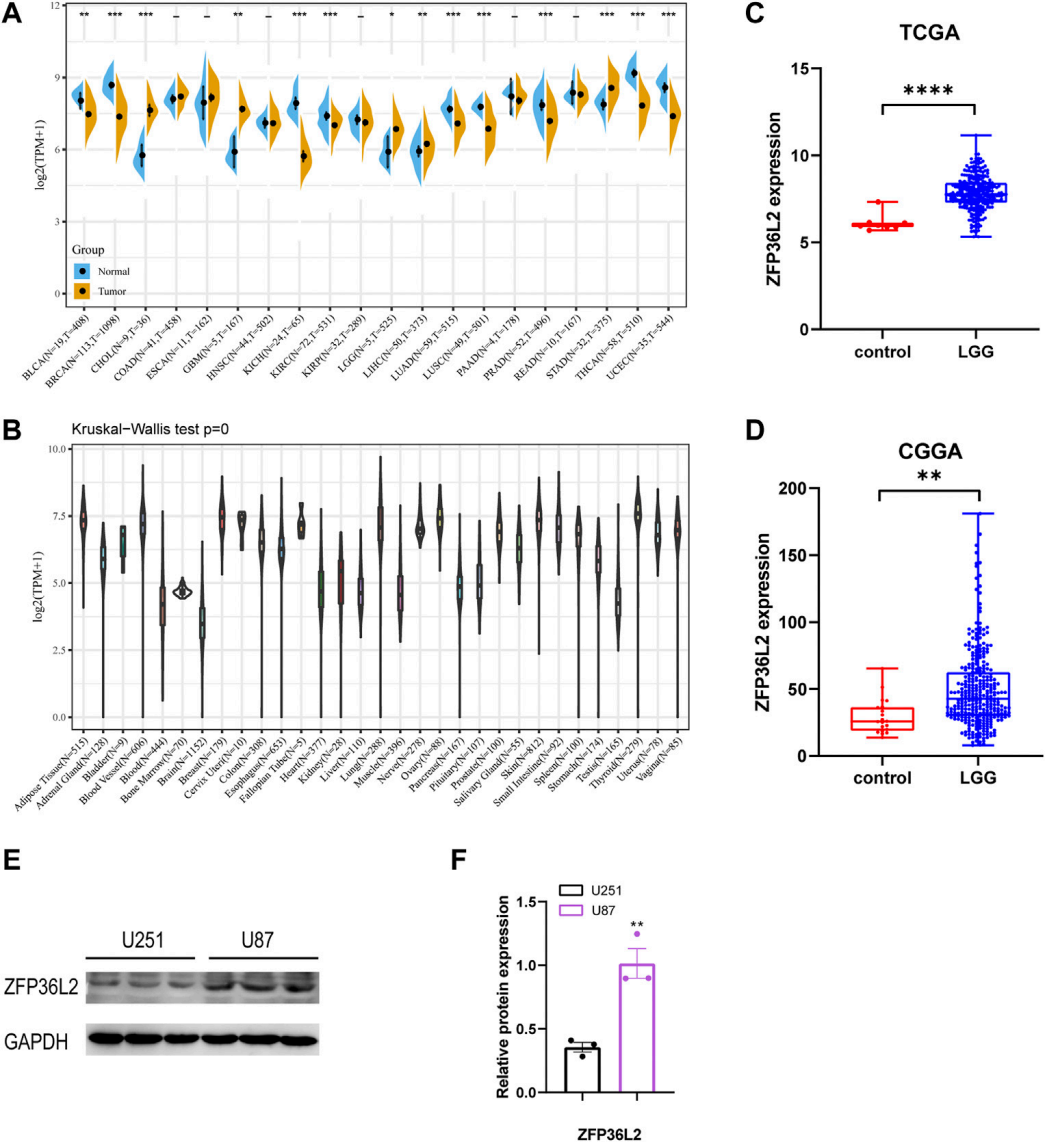
Expression levels of ZFP36L2 across different types of tumor and normal tissues. **(A)**The data from UCSC database showed the expression of ZFP36L2 in different tumor tissues and corresponding normal tissues. **(B)** The expression of ZFP36L2 in various normal tissues. **(C,D)** ZFP36L2 mRNA is highly expressed in LGG tissues in TCGA dataset and CGGA dataset. **(E,F)** Protein level analysis of ZFP36L2 in U251 and U87 cell lines by western blot; GAPDH was used as a loading control (*n* = 3).**p < 0.01, two-tailed unpaired Student’s t-test. BLCA, Bladder Urothelial Carcinoma; BRCA, Breast invasive carcinoma; CHOL, Cholangiocarcinoma; COAD, Colon adenocarcinoma; ESCA, Esophageal carcinoma; GBM, Glioblastoma multiforme; HNSC, Head and Neck squamous cell carcinoma; KICH, Kidney Chromophobe; KIRC, Kidney renal clear cell carcinoma; KIRP, Kidney renal papillary cell carcinoma; LGG, Brain Lower Grade Glioma; LIHC, Liver hepatocellular carcinoma; LUAD, Lung adenocarcinoma; LUSC, Lung squamous cell carcinoma; PAAD, Pancreatic adenocarcinoma; PRAD, Prostate adenocarcinoma; READ, Rectum adenocarcinoma; STAD, Stomach adenocarcinoma; THCA, Thyroid carcinoma; UCEC, Uterine Corpus Endometrial Carcinoma.

We initially evaluated ZFP36L2 mRNA expression levels in different human tumor and normal tissues based on TCGA TARGET GTEx cohort. The result showed that ZFP36L2 was highly expressed in colorectal cancer, lower-grade gliomas, leukemia, hepatic cancer, ovarian cancer, pancreatic cancer and gastric cancer compared with normal tissues. However, in breast cancer, lung cancer, melanoma and cervical cancer, the expression of ZFP36L2 was low (Figure 1A). Interestingly, based on the Human Protein Atlas (HPA) database (Sjöstedt et al., 2020), we found brain tissue had the lowest ZFP36L2 expression across different types of human tissues (Figure 1B). To further confirm the expression of ZFP36L2 in LGG, we analyzed two credible cohorts, and found the expression of ZFP36L2 was significant higher in LGG (Figures 1C,D). Furtherly, increased levels of ZFP36L2 were detected by western blot in a more malignant glioma cell line, U87, indicating ZFP36L2 might be associated with glioma development at least in part. Overall, the above findings indicated that ZFP36L2 was highly expressed in LGG, and dysregulated expression of ZFP36L2 might predict poor prognosis of LGG.

### 3.2 Increased ZFP36L2 expression is correlated with poor survival

**FIGURE 2:**
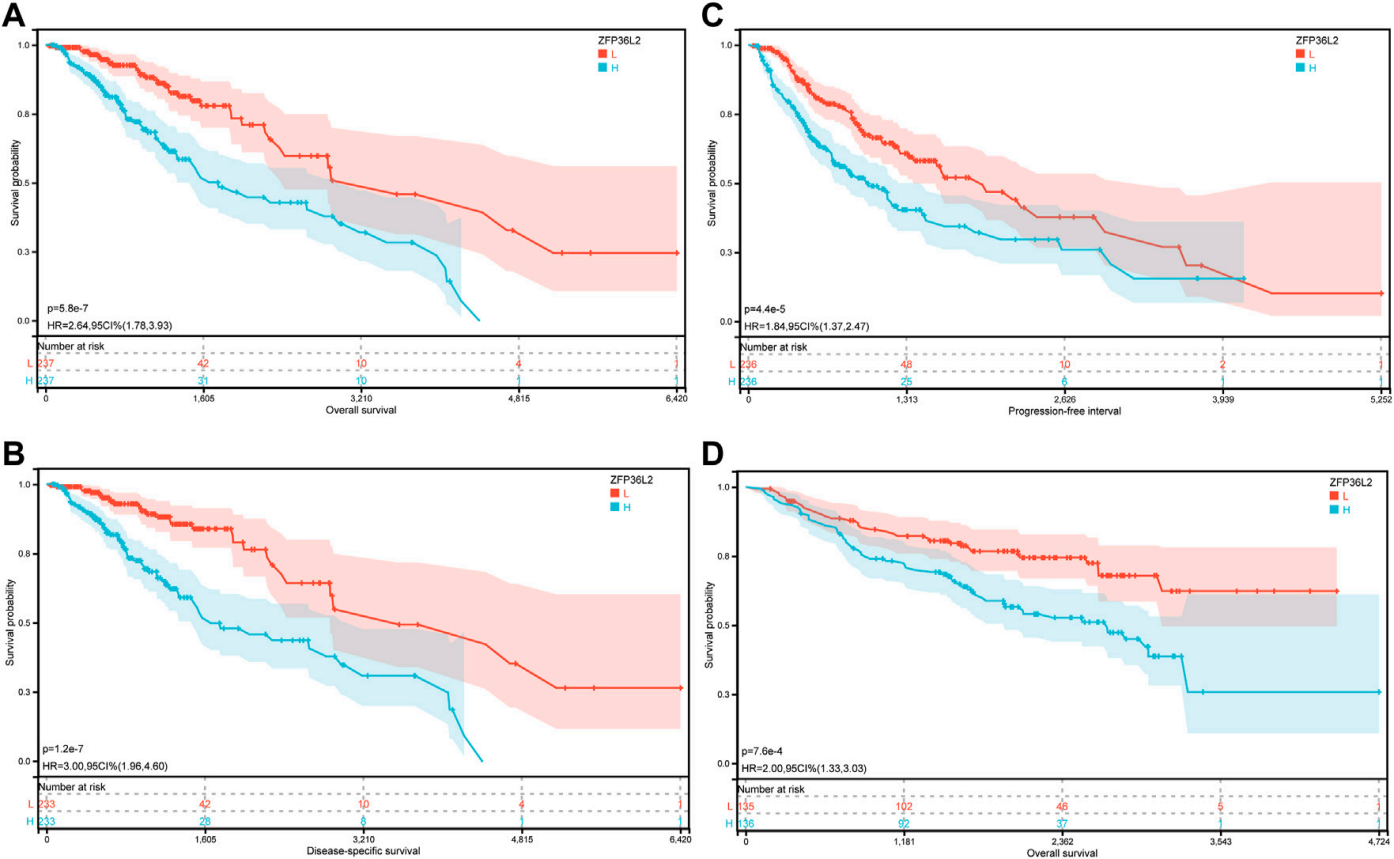
The prognostic values of ZFP36L2 expression in LGG. **(A)** Overall survival curve of ZFP36L2 in TCGA-LGG (*n* = 445). **(B)** Disease-specific survival curve of ZFP36L2 in TCGA-LGG (n = 466). **(C)** Progression-free survival curve of ZFP36L2 in TCGA-LGG (n = 466). **(D)** Overall survival curve of ZFP36L2 in CGGA-LGG.

**FIGURE 3:**
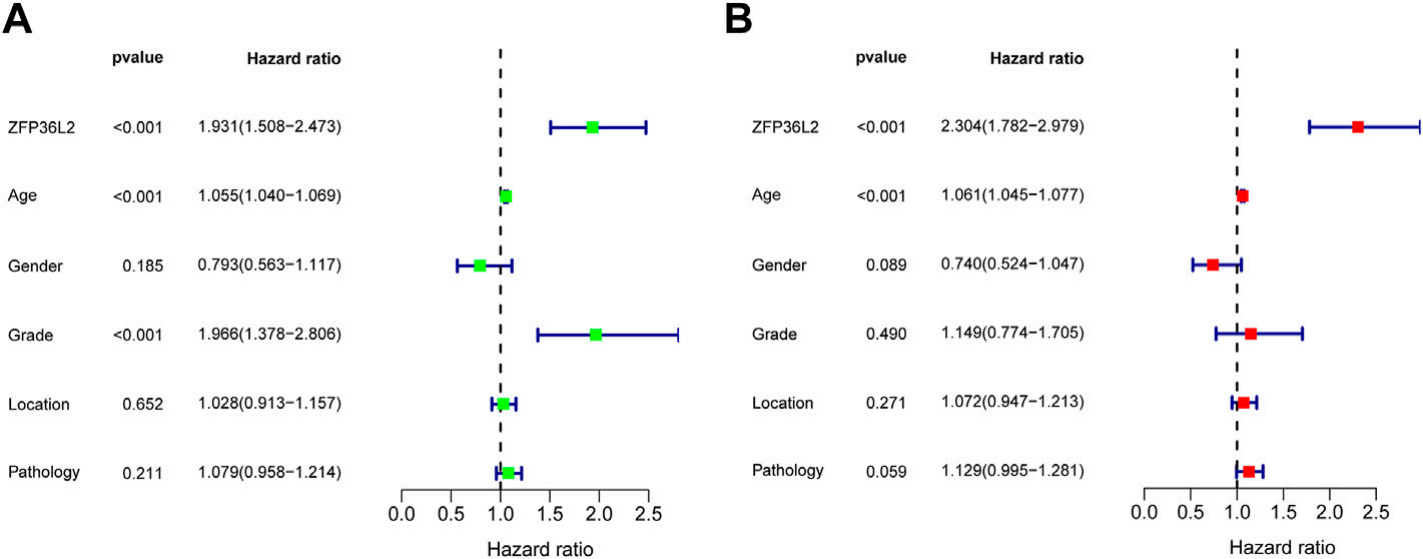
Univariate and Multivariate Analysis. Univariate **(A)** and Multivariate **(B)** Analysis indicated that expression level of ZFP36L2 was significantly associated with overall survival (*p*-value < 0.001).

Given the sharp contrast of ZFP36L2 expression between tumor and normal tissues, we further evaluated the prognostic role of ZFP36L2 in LGG based on two independent cohorts, the TCGA and CGGA databases. Kaplan-Meier survival analysis was performed and the data showed that high expression of ZFP36L2 predicted shorter overall survival (OS), progression-free survival and disease-specific survival (Figures 2A–C). Consistently, the association between ZFP36L2 and patient outcomes was also verified in CGGA cohort. Then, we introduced Cox Regression Analysis to figure out whether ZFP36L2 is an independent biomarker in LGG. Univariate analysis indicated that age, grade and ZFP36L2 expression were significantly correlated with overall survival. Furthermore, Multivariate Cox Analysis, after adjusting additional clinical relevant factors, revealed that ZFP36L2 expression was an independent predictor in LGG patients (Figures 3A,B).

Hypomethylation is associated with ZFP36L2 upregulation and predicts poorer prognosis in LGG.

**FIGURE 4:**
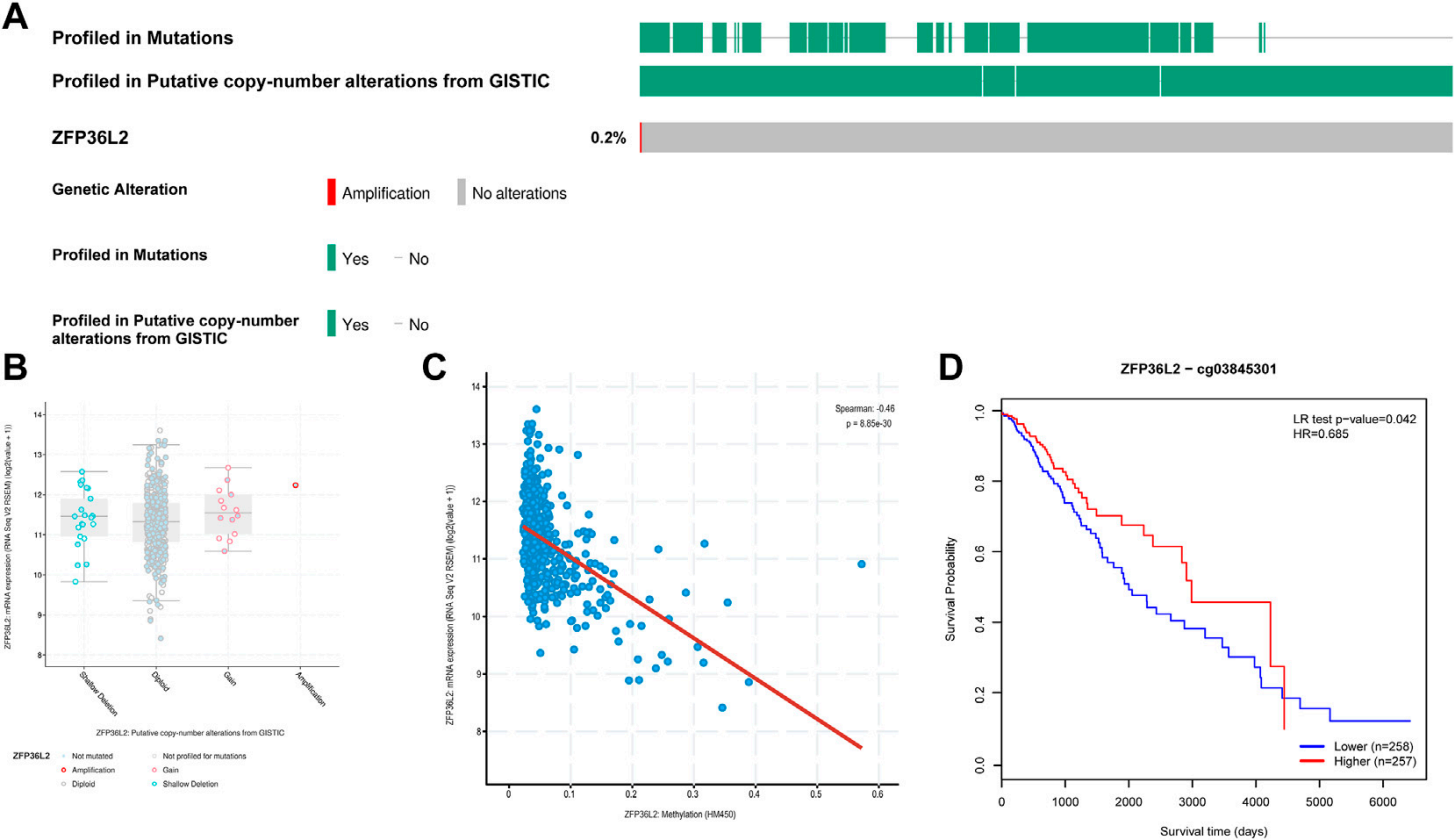
Genetic alterations of ZFP36L2 expression in low-grade gliomas. **(A)** Mutation rate of ZFP36L2 in LGGs. **(B)** Putative copy number alterations of ZFP36L2 in LGGs. **(C)** The correlation between ZFP36L2 methylation and its expression level. **(D)** The Kaplan-Meier survival of the methylation of SEC61G in LGG.

In view of the significant association between ZFP36L2 overexpression and the development of glioma, we further explored the mechanism of ZFP36L2 upregulation in glioma. Using cBioPortal, we found that ZFP36L2 was not mutated in LGG and only a very small number of patients were accompanied by the amplification of CNV of ZFP36L2 (Figures 4A,B). This suggests that genetic changes may not be the main cause of ZFP36L2 upregulation in gliomas. We further analyzed the relationship between ZFP36L2 methylation and its mRNA expression, and these results showed that gene methylation negatively related with ZFP36L2 gene expression (R = −0.46, *p* < 0.001) (Figure 4C). Furthermore, the MethSurv analyses indicated that patients with lower levels of ZFP36L2 methylation have a poorer prognosis.

### 3.3 Distinct ZFP36L2 expression pattern characterized by biological enrichment analysis

**FIGURE 5:**
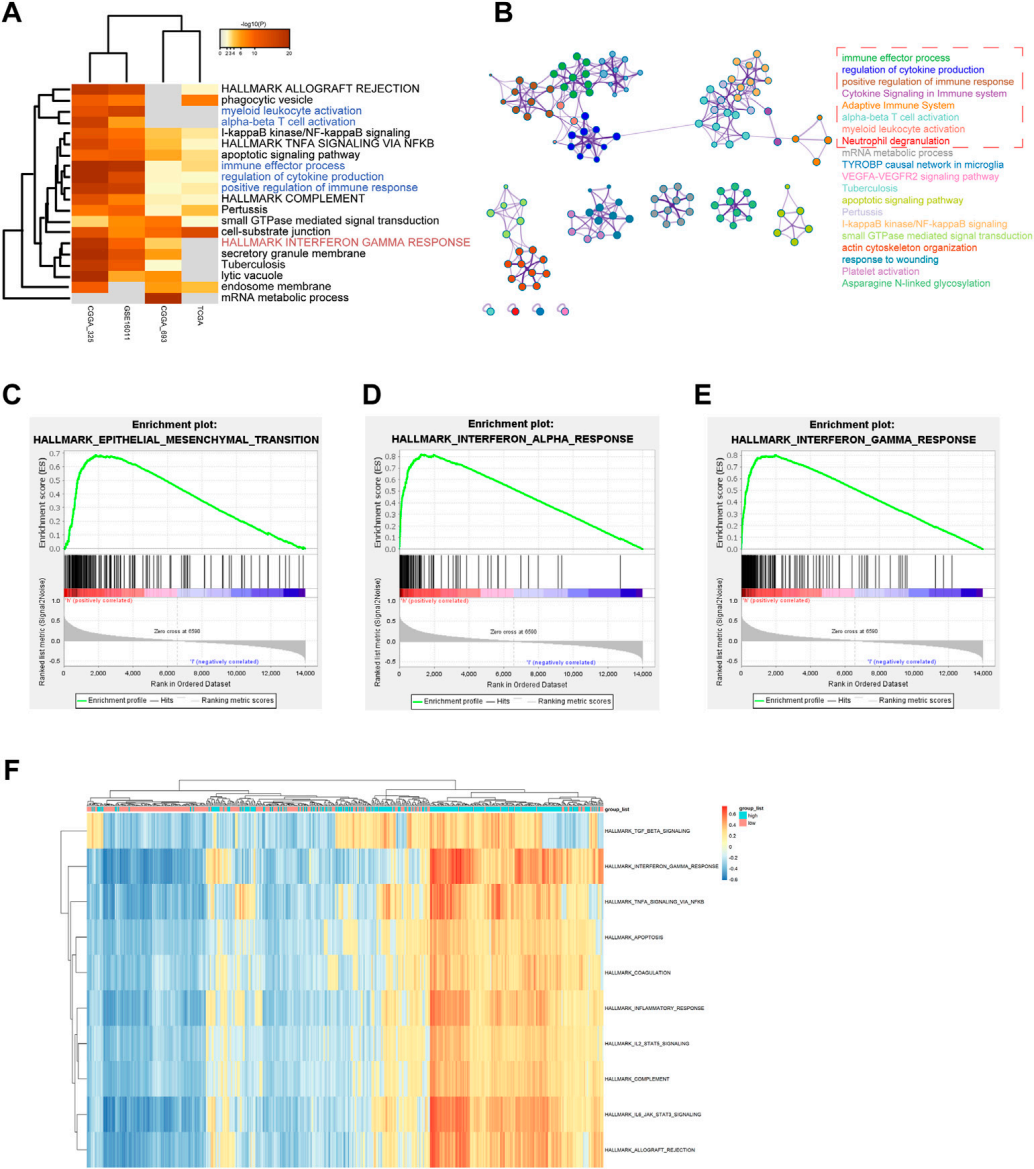
Distinct ZPF36L2 expression pattern characterized by biological enrichment analysis. **(A)** Functional enrichment analysis of ZFP36L2 co-expression genes based on different LGG datasets. **(B)** Network of enriched terms of ZFP36L2 co-expression genes. The enrichment networks colored by cluster IDs. Each color represents a class of clusters, and each point represents a term. The immune-related terms are selected in the red dashed box. **(C–E)** The GSEA method was used to explore the biological functions that were enriched in ZFP36L2 high expression patients. **(F)** Heatmap shows the GSVA score of representative hallmark pathways in distinct ZFP36L2 expression patterns.

To explore the ZFP36L2-related biological characteristics in LGG, Spearman’s Correlation Analysis was used to identify ZFP36L2 co-expressed genes in four independent datasets from all samples. Metascape (Zhou et al., 2019) was further used to examine the biological characteristics of ZFP36L2 co-expressed genes (Figures 5A,B). The results identified the statistically enriched terms across all LGG datasets, including alpha-beta T cell activation, positive regulation of immune response and interferon gamma response. GSEA and GSVA analysis were also performed to examine the underlying biological molecular changes between ZFP36L2-high and -low expression cohort. GSEA analysis shown that several immune and tumor-related terms were enriched in ZFP36L2-high expression cohort, including epithelial mesenchymal transition, interferon alpha response and interferon gamma response (Figures 5C–E). Furthermore, GSVA analysis indicated that inflammatory response and interferon gamma response were enriched in group with ZFP36L2 high expression (Figure 5F).

### 3.4 Relationship between ZFP36L2 expression and immune infiltration in LGG

**FIGURE 6:**
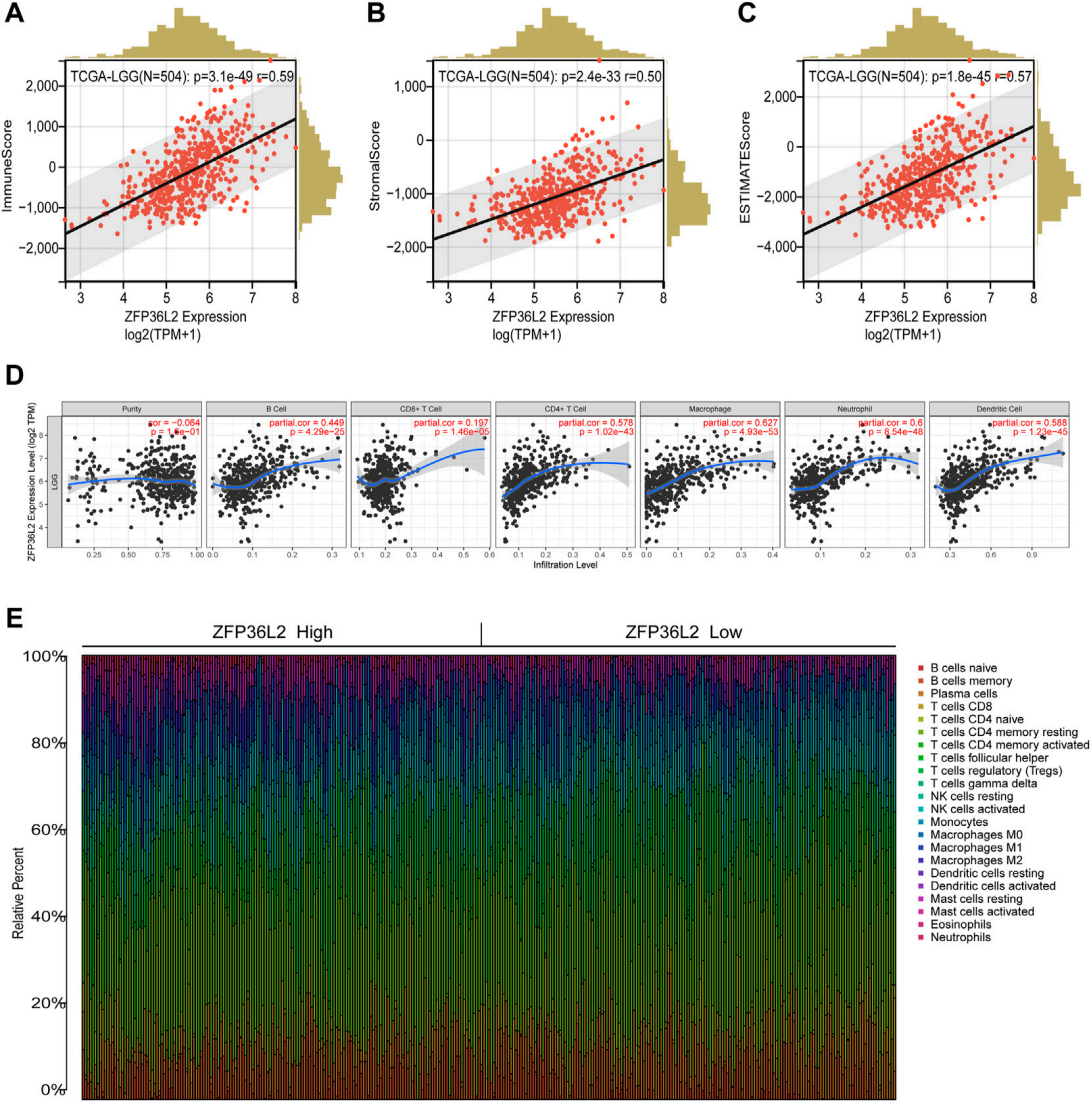
Relationship between ZFP36L2 expression and immune infiltration in LGG. **(A–C)** ZFP36L2 expression positively correlated with immune score, stromal score and ESTIMATE score in LGG. **(D)** ZFP36L2 expression is significantly positively related to infiltrating levels of B cells, CD8^+^ T cells, CD4^+^ T cells, macrophages, neutrophils, and dendritic cells in LGG. **(E)** The heatmap visualized the percentage abundance of tumor-infiltrating immune cells in each sample.

The tumor microenvironment plays an important role in tumor outcome. Thus, we estimated the non-tumor cell infiltration degree involved in tumor microenvironment of LGG patients by using ESTIMATE algorithm. The results indicated that ZFP36L2 expression was significantly positively associated with Immune Score, Stromal Score and Estimate Score (Figures 6A–C). We further examined whether ZFP36L2 expression was related with different types of immune cells based on the TIMER database. The result showed that the expression of ZFP36L2 was significantly positively correlated with the main immune cells infiltration degree (Figure 6D). Finally, we visualized the proportion of individual immune cells in each sample of ZFP36L2 high- and low-group, respectively (Figure 6E).

### 3.5 The correlation between ZFP36L2 expression and immunotherapy response

**FIGURE 7:**
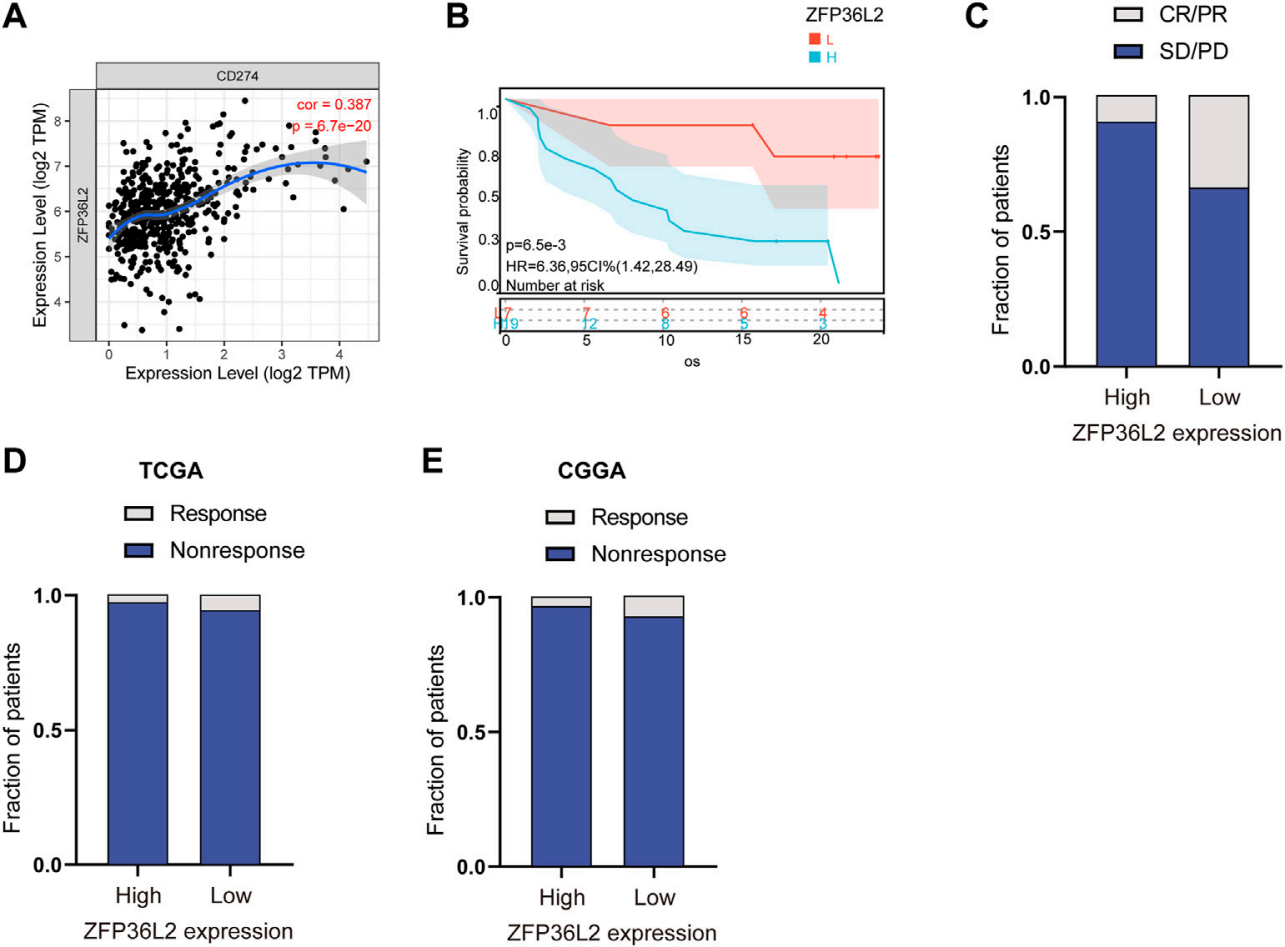
The correlation between ZFP36L2 expression and immunotherapy response. **(A)** The positively expressing correlation between ZFP36L2 and PD-L1. **(B)** Overall survival curve of ZFP36L2 high- and low-expression groups in anti-PD-L1 cohort (IMvigor210) treated with atezolizumab. **(C)** The proportion of patients with response to PD-L1 blockade immunotherapy in low or high ZFP36L2 expression groups. SD, stable disease; PD, progressive disease; CR, complete response; PR, partial response. Responser/Nonresponser: 8.3%/91.7% in the ZFP36L2 high-expression groups and 33%/67% in the ZFP36L2 low-expression groups. **(D)** The predictive results of “ImmuneCellAI” in TCGA cohort. Response/Nonresponse: 8/264 in the ZFP36L2 high-expression groups and 17/265 in the ZFP36L2 low-expression groups. **(E)** The predictive results of “ImmuneCellAI” in CGGA_693 cohort. Response/Nonresponse: 5/141 in the ZFP36L2 high-expression groups and 11/141 in the ZFP36L2 low-expression groups.

Immunotherapy represented by anti-PD-L1 is widely used in tumor treatment. We first assessed the correlation between ZFP36L2 and PD-L1 expression, and the results showed that ZFP36L2 was significantly positively correlated with PD-L1 (Figure 7A). The anti-PD-L1 cohort (IMvigor210) (Balar et al., 2017) treated with atezolizumab was utilized to investigate the relationship between ZFP36L2 and patients’ response to immunotherapy. The results showed that patients with low ZFP36L2 expression had significantly prolonged survival (Figure 7B). Furthermore, the proportion of patients with better efficacy for immunotherapy in the ZFP36L2 low-expression group was significantly higher compared to the high-expression group (Figure 7C). Finally, we used “ImmuneCellAI” analyses to evaluated the patient response to ICIs. The results shown that the proportion of patients who responded to ICIs in the ZFP36L2 low-expression group was higher than that in the ZFP36L2 high-expression group in both TCGA and CGGA cohort (Figures 7D,E).

## 4 Discussion

RNA-binding protein, a key player of post-transcriptional regulation of gene expression, is becoming a major driving factor of tumor initiation and progression. ZFP36L2 belongs to the zinc finger protein family and plays an opposite role in different types of cancer. However, the impact of ZFP36L2 on LGG clinical prognosis remains unknown. Here, we performed a comprehensive bioinformatic analysis to study the potential relationship between ZFP36L2 and LGG.

In this study, we first confirmed that, compared with normal brain tissue, ZFP36L2 expression was significantly higher in LGG. Interestingly, the brain has the lowest expression across different types of tissues. The strong contrast of ZFP36L2 expression between LGG and normal brain tissue suggests that it may play a non-negligible role in the development of LGG. Thus, we further examined whether ZFP36L2 was an independent prognostic factor in LGG patients. Various clinical factors were included to perform univariate and multivariate regression analyses, such as age, gender, stage, location and pathology, and the results showed that ZFP36L2 was significantly associated with prognosis after excluding these clinical factors, suggesting that ZFP36L2 was a reliable biomarker of LGG prognosis.

Considering the strong prognostic value of ZFP36L2 overexpression, we further explored the mechanism of ZFP36L2 upregulation in glioma. DNA methylation and CNVs are two widely known reasons contributing to increased gene expression. Genetic alteration is a hallmark of cancers. Xing et al. used whole-exome sequencing to identify a novel hotspot involving a super-enhancer of ZFP36L2, which drives ZFP36L2 overexpression in gastric cancer (Xing et al., 2019). Our results suggest that the ZFP36L2 expression in DNA amplification group is elevated, but only a very small number of patients exhibited this (Figure 4B). Genetic alteration may not be a major driver of ZFP36L2 upregulation. DNA methylation is an epigenetic regulatory mechanism widely present in all types of tumors, and methylation of promoters leads to gene silencing. Our results demonstrated that ZFP36L2 overexpression significantly associated with ZFP36L2 hypomethylation. More importantly, ZFP36L2 hypomethylation had a worse prognosis, consistent with the prognostic value of its mRNA overexpression. Overall, hypomethylation might be the main regulatory mechanism of ZFP36L2 overexpression.

To gain a deeper understanding of the biological processes involved in the development of LGG related to ZFP36L2 expression, we used ZFP36L2 co-expressed genes to perform functional enrichment analysis. Our results suggested that ZFP36L2-related genes were mainly involved in immune response, immune cell activation and cytokine production. Furthermore, GSEA and GSVA enrichment analyses were performed to finger out the biological changes between ZFP36L2 high-expression and low-expression groups. The data revealed that ZFP36L2 high-expression cohort was significantly enriched in interferon gamma response, TGF-β, epithelial mesenchymal transition, and allograft rejection. To our knowledge, dysregulated immune response and cytokine production play an important role in tumor invasion, recurrence and metastasis (Fridman et al., 2012; Gajewski et al., 2013; Tanaka and Sakaguchi, 2016; Fang et al., 2019). These findings indicated that ZFP36L2 might be involved in the development of LGG by regulating the tumor immune microenvironment.

The tumor microenvironment is composed of immune cells, stromal cells and extracellular components, which play an important role in the initiation, development and metastasis of tumors (Hanahan and Coussens, 2012; Quail and Joyce, 2013). Given that ZFP36L2 is involved in multiple immune-related pathways, we further evaluated its role in the tumor immune microenvironment. ESTIMATE algorithm and TIMER database were utilized to evaluate the correlation between ZFP36L2 expression and immune infiltration level in the tumor microenvironment of LGG samples. The results showed that ZFP36L2 was significantly positively correlated with the three immune scores and multiple types of immune cells, while immune cells had been demonstrated being significantly related to the progression of glioma in previous studies (Patel et al., 2014; Wang et al., 2018). Collectively, our findings provide new insights into the relationship between the tumor immune microenvironment and glioma.

Immunotherapy, represented by targeting immune checkpoints, has attracted great attention recently. However, the efficacy of immunotherapy for some patients with LGG did not meet expectations (Xu et al., 2020). Considering the strong correlation between ZFP36L2 expression and immune response and tumor microenvironment, we further evaluated its relationship with immunotherapy response. Interestingly, expression of ZFP36L2 was significantly positively correlated with that of PD-L1, and in an anti-PD-L1 cohort, patients with low ZFP36L2 expression had better clinical outcomes. Furthermore, “ImmuneCellAI” analyses were performed to evaluated the patient response to ICIs, and the results indicated that ZFP36L2 low-expression group benefited more. These results suggest that ZFP36L2 could be recognized as a predictive biomarker for response to immunotherapy.

Although this study improved our understanding of ZFP36L2 in LGG, there were some limitations. First, the prognostic value of ZFP36L2 for different subtypes of LGG patients is unknown. Based on WHO 2016 Classification of gliomas, LGG was classified into three subtypes according to IDH mutation and 1p/19q co-deletion status. The prognostic value of ZFP36L2 overexpression in different subtypes of LGG needs further evaluation. Second, the detailed mechanisms of involved in glioma development is not clear. While we found that ZFP36L2 is positively correlated with immune cell infiltration and a better response is presented in ZFP36L2 low-expression group, the conclusion is based only on bioinformatics analysis. *In vivo* and *in vitro* experiments are needed to verify this conclusion. Thus, we will continue to explore the mechanism of ZFP36L2 involved in glioma development in a future study.

In this study, our finding indicated that ZFP36L2 was significantly increased in LGG and patients with higher ZFP36L2 expression had poorer overall survival. We also determined the correlation between ZFP36L2 expression and genetic changes. In addition, ZFP36L2 mediates multiple immune response pathways and is involved in the regulation of the tumor microenvironment. Taken together, the comprehensive assessment of the role of ZFP36L2 in LGG will help to enhance our understanding of the development of LGG and provide guidance for immunotherapy with LGG patients.

## Data Availability

The original contributions presented in the study are included in the article/supplementary material, further inquiries can be directed to the corresponding authors.
